# A systematic review and meta-analysis of the seroprevalence of *Toxoplasma gondii* in cats in mainland China

**DOI:** 10.1186/s13071-017-1970-6

**Published:** 2017-01-13

**Authors:** Huan Ding, Yu-Meng Gao, Yao Deng, Poppy H. L. Lamberton, Da-Bing Lu

**Affiliations:** 1 Department of Epidemiology and Statistics, School of Public Health, Soochow University, Suzhou, 215123 China; 2 Jiangsu Key Laboratory of Preventive and Translational Medicine for Geriatric Diseases, School of Public Health, Soochow University, Suzhou, 215123 People’s Republic of China; 3 Institute of Biodiversity, Animal Health and Comparative Medicine and Wellcome Trust Centre for Molecular Parasitology, University of Glasgow, Glasgow, GL12 8QQ UK; 4 Department of Infectious Disease Epidemiology, Imperial College London, London, W2 1PG UK

**Keywords:** *Toxoplasma gondii*, Cats, Mainland china, Seroprevalence, Meta-analysis

## Abstract

**Background:**

Toxoplasmosis is caused by *Toxoplasma gondii* which can infect all warm-blooded animals. As the most common feline definitive host, cats play a vital role in the transmission of *T. gondii*. However, national estimates of the seroprevalence of *T. gondii* in cats in mainland China are lacking, and therefore a systematic review and meta-analysis were performed to provide insight into national environmental transmission levels and potential transmission to humans.

**Methods:**

Studies published up until July 1, 2016, on *T. gondii* seroprevalence in cats within mainland China were searched for in CNKI, WanFang, CBM, PubMed, Embase and through the reference lists of resulting articles. The seroprevalence with its 95% confidence interval (CI) for each individual study was presented, and then point estimates and their 95% confidence intervals (CIs) of pooled seroprevalence were calculated. Subgroup analyses were performed according to potential risk factors.

**Results:**

A total of 38 eligible studies, published between 1995 to 2016, covering fifteen provinces and municipalities, and involving 7,285 cats, were included. The seroprevalence in cats per study ranged from 3.9 to 79.4% with a median of 20.3%. As substantial heterogeneity existed among studies, a random-effects model was used to estimate the pooled seroprevalence. The value of the point estimate seroprevalence was 24.5% (95% CI: 20.1–29.0). Seroprevalence in stray cats was significantly higher than in pet cats (OR = 3.00, 95% CI: 1.60–5.64). The seroprevalence increased significantly with cat age (*P* = 0.018) with 17.4% (95% CI: 7.6–27.2) in the group of ≤ 1 year old, 19.5% (95% CI: 12.7–26.3) in the group of ≤ 3 year-old and 31.6% (95% CI: 22.9–40.3) in the group of > 3 year-old.

**Conclusions:**

The seroprevalence of *T. gondii* in cats in mainland China was moderate and was associated with cat ownership and age. Due to the increasing prevalence of pet cats in China and the intimate relationship between these cats and humans, this might present a significant exposure risk, particularly for China’s large susceptible population. Therefore, further research is needed into the links between cat ownership and human *T. gondii* infection and how to reduce *T. gondii* exposure in humans *via* cat contacts and the environmental contamination with *T. gondii* oocysts by cats.

**Electronic supplementary material:**

The online version of this article (doi:10.1186/s13071-017-1970-6) contains supplementary material, which is available to authorized users.

## Background

Toxoplasmosis is caused by the obligate, intracellular protozoan *Toxoplasma gondii*, a widespread zoonotic parasite which can infect all warm-blooded animals [1], and is one of the most common zoonosis in the world [2]. Its wide distribution may be attributed to complex transmission patterns and parasite coevolution with multiple hosts [3]. Felids are the only definitive host and one infected cat can discharge millions of infective oocysts in faeces, although only over a few days after primary infection [4, 5]. Intermediate hosts (such as humans, rodents and other animals) can be infected through ingestion of oocysts from the environment (food contaminated with oocysts or direct contact with oocysts excreted in cats faeces), consumption of undercooked meat containing *T. gondii* tissue cysts [6, 7], or congenitally when parasites in a pregnant women infected with *T. gondii* for the first time spread to the foetus through the placenta often causing abortion, premature birth, stillbirth, malformation and/or neonatal congenital infection [8].

Although *T. gondii* infections of immunocompetent people are typically considered asymptomatic, infections in immunocompromised individuals, such as those with AIDS or organ transplant recipients, can result in severe consequences. For example, approximately 10% of AIDS patients in the USA and up to 30% in Europe are estimated to die from toxoplasmosis [9]. Moreover, positive correlations between previously assumed asymptomatic *T. gondii* infections with the incidences of schizophrenia [10], car accident [11], epilepsy [12] and suicide [13] in humans have now been reported. The seroprevalence of toxoplasmosis in psychiatric patients was once reported to be as high as 50% [11]. Globally, in 2010 *T. gondii* was estimated to have caused 10.28 million foodborne illnesses and 0.83 million Disability Adjusted Life Years (DALYs) [14]. These all highlight the global public health importance of this infection in human populations.

Toxoplasmosis remains a public health problem in mainland China, as there is an increasing number of AIDS patients with an estimate of 650,000 in 2005 increasing to 780, 000 in 2011 [15] and a huge number of women of childbearing age, estimated to be approximately 375.8 million in 2013 [16]. Cats play a major role in the transmission of *T. gondii*, pet cats may therefore be an important potential source of human toxoplasmosis due to their intimate association with humans, particularly if they are free-roaming and may themselves be exposed to environmental *T. gondii* parasites. The seroprevalence of *T. gondii* in pet cat owners (11.86%) is higher than in non-pet cat owners (7.38%) [17] or than in the general population (7.88%) surveyed in 2001–2004 [18], and the seroprevalence in some areas of China was as high as 34% [19]. With the rapid development of the Chinese economy and continuous improvement of living standards in China the number of families which have pet cats is increasing. For example, it was estimated that approximately 100 million cats were considered pets in 2010 in China [20], with a growth rate of 10% over the subsequent years [21].

To the authors’ knowledge, there is no study which has addressed the overall seroprevalence of *T. gondii* infection in cats across mainland China nor the risk factors associated with these infections. Therefore, this systematic review and meta-analysis was performed to determine the seroprevalence of *T. gondii* in cats in mainland China over the last 20 years and to assess the potential risk factors related to *T. gondii* seroprevence in cats. The purpose was to provide an increased understanding to aid parasite control, as evidence grows of its importance for human health [22, 23], particularly in China with such a large susceptible population.

## Methods

The study was conducted according to the PRISMA guideline (Preferred Reporting Items for Systematic Reviews and Meta-Analyses) [24]. The PRISMA checklist was used to ensure inclusion of relevant information in the analysis (see Additional file 1).

### Search strategy

A literature search was conducted for publications from January 1, 1995 to July 1, 2016. We aimed to include all published studies in English or Chinese on seroprevalence of *T. gondii* in cats across mainland China. We identified published studies within the following five bibliographic databases (three in Chinese and two in English): “*toxoplasma gondii*” and “*cat*” in Chinese (“gongxingchong/or gongxingti”, and “mao”, respectively) were used as search terms in the Chinese databases (China National Knowledge Infrastructure (CNKI), WanFang and The Chinese Biomedical Literature Database (CBM)), and “*toxoplasma*” and “china” and “cats” were MeSH terms in the PubMed online and “*toxoplasma*” and “china” and “cats” were emtree term-exploded in Embase. We also visually scanned all reference lists from relevant studies in an attempt to locate additional studies that may not have been identified by searching the electronic databases. We did not contact authors of original studies for additional information. No attempt was made to retrieve unpublished studies. Full text articles were downloaded or obtained through library resources.

### Inclusion and exclusion criteria

**Fig. 1:**
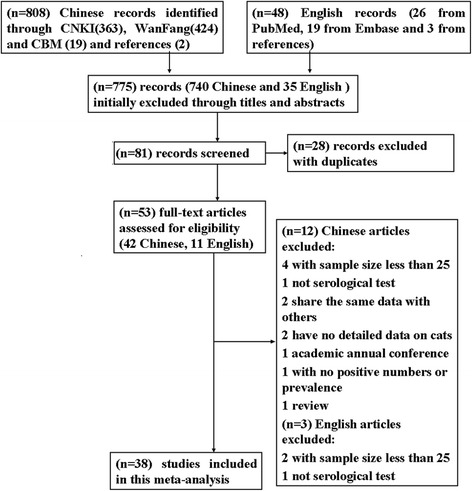
Flow diagram of the selection of eligible studies

A total of 53 full texts were read for eligibility screening (Fig. 1). Selected manuscripts needed to fulfil the following inclusion criteria: (i) cross-sectional study; (ii) locations within mainland China; (iii) targeted objectives included cats; (iv) serological diagnostic methods were used; (v) exact total and positive numbers were provided; and (vi) a sample size greater than 25 (for statistic calculations [25]). Studies were excluded if they did not fulfil all of these criteria.

### Quality of the studies

We evaluated risk of bias among the included studies using a quality assessment checklist. The following items were examined and given a score based on a simple scale system (2 for “yes”, 0 for “no”, or 1 for “unsure”).

- Was the research question/objective clearly described and stated?
- Was the sampling method described in detail?
- Was the period of study clearly stated?
- Was the serological test method clearly pointed out?
- Were the subjects categorized into different subgroups?

### Data extraction

For data extraction, the detailed characteristics of each study were extracted using a pre-designed data-collection excel form. Information was recorded as follows: study characteristics (the first author, year of publication, year of study, location); study methodology (survey method in detail, sampling method and the serological test used); characteristics of cats (pet or stray, gender, age category, survey season and region); sample size; the number of the positives and/or seroprevalence of *T. gondii*; score of each study.

### Data analysis

While the inverse variance method is widely used and works for prevalence proportions around 0.5, two problems arise when the proportions get closer to the limits of the 0 and 1 range. The first of these problems is that the confidence interval (CI) does not preclude confidence limits outside the 0–1 range; the second problem is that a study gets a large weighting when the proportion becomes too small or too big [26]. Therefore, we here calculated seroprevalence estimates with the variance stabilising double arcsine transformation by the following formula: t = arcsin (sqrt (r/(*n* + 1))) + arcsin (sqrt ((r + 1)/(*n* + 1))), where t = transformed seroprevalence, r = positive numbers and *n* = sample size; se(t) = sqrt(1/(*n* + 0.5)), where se = standard error and the back transformation to a proportion is done using: *p =* (sin(t/2))^2^ [26].

### Pooling and heterogeneity analyses

The seroprevalence and its 95% CI for each study were first calculated, and then point estimates and their 95% CIs of pooled seroprevalence of all included studies were analyzed. Forest plots were used to express the results of each study and the heterogeneity among studies. Summary of seroprevalence estimates were obtained using fixed-effects or random-effects meta-analyses which were determined by the I^2^ statistic (inverse variance index), which describes the percentage of variation between studies that is due to heterogeneity rather than chance. I^2^ does not inherently depend upon the number of studies considered, with values of 25, 50 and 75% corresponding to low, moderate, and high degrees of heterogeneity, respectively [27].

Potential sources of heterogeneity were investigated further by arranging groups of studies according to potentially relevant characteristics. In this study, subgroup analysis was stratified by group (i.e. stray or pet), gender (male or female), age (≤ 1 year, > 1 year ≤ 3 years, or > 3 years), geographical regions (Eastern region including: Beijing, Tianjin, Hebei Province, Liaoning Province, Shanghai, Jiangsu Province, Zhejiang Province, Fujian Province, Shandong Province, Guangdong Province and Hainan Province; Central region including: Shanxi Province, Jilin Province, Helongjiang Province, Anhui Province, Jiangxi Province, Henan Province, Hubei Province and Hunan Province; or Western region including: Sichuan Province, Chongqing, Guizhou Province, Yunnan Province, Tibet Autonomous Region, Shanxi Province, Gansu Province, Qinghai Province, Ningxia Hui Autonomous Region, Xinjiang Uygur Autonomous region, Guangxi Zhuang Autonomous Region and Inner Mongolia Autonomous Region), survey seasons (Spring, Summer, Autumn and Winter), and main serological tests. Meta-regression was used to investigate any significant difference between/among subgroups and the value of an odds ratio was calculated.

### Bias and sensitivity tests

The across-study bias (publication bias) was examined by funnel plots. In addition, the statistical significance was assessed by the Egger’s regression asymmetry test [28] and Begg rank correlation method [29]. The Duval & Tweedie non-parametric ‘fill and trim’ linear random method was used to test and adjust for publication bias [30]. To test the robustness of a pooled estimate, we evaluated the effect of each study on the pooled seroprevalence by excluding single studies sequentially (i.e. estimated based on 37 studies each time). A study was deemed to have no influence if the pooled estimate without it (i.e. *n* = 37) was within the 95% confidence limits of the overall seroprevalence (*n* = 38) [31].

Extracted data were entered into Microsoft Office Excel 2007 and Stata 12.0 was used in all statistical analyses.

## Results

### Search results and eligible studies

We retrieved 856 published studies through five databases and the reference lists of relevant studies (Fig. 1). A total of 775 records were excluded through an initial screening of the titles and/or abstracts. A further 28 records were excluded when taking duplication into account. The remaining 53 full-text articles were assessed, of which 15 records were further excluded according with our inclusion criterion. A total of 38 studies [32–69] were included in this meta-analysis.

### Characteristics of the eligible studies

Table 1 shows the characteristics of the final 38 studies eligible for inclusion, which covered 15 provinces and municipalities. The years of the studies performed and published ranged from 1991 to 2015 and from 1995 to 2016, respectively. The total number of cats was 7,285, with a range of 27 to 589 per study. Serological assays used in eligible studies retrieved only involved four tests including Enzyme Linked Immunosorbent Assay (ELISA, *n* = 22), Indirect Hemagglutination Test (IHA, *n* = 10), Modified Agglutination Test (MAT, *n* = 4), and Test Paper (*n* = 2). The evaluated scores indicating the quality of selected studies were from 6 to 10.

**Table 1:** Characteristics of the eligible studies

Author	Year	Region	Period of study	Serological method	Positivity	Detailed information on cats	Total no. of cats	No. of positive cats (%)	Quality score
Fu et al. [36]	1995	Shandong	1991–1993	IHA^a^	≥ 1:64	No	200	92 (46.00)	8
Lu et al. [41]	1997	Shanghai	1994–1995	IHA^b^	≥ 1:80	Gender, age, Season	142	54 (38.01)	10
Chen et al. [32]	2001	Hubei		ELISA^f^	IgG or CAg positive	No	105	33 (31.43)	8
Zhao et al. [58]	2001	Shandong		IHA^a^	≥ 1:64	No	185	82 (44.32)	7
Chen et al. [66]	2003	Shenzhen, Guangdong		IHA^a^	≥ 1:64	No	65	12 (18.46)	6
Yuan et al. [54]	2004	Baoding, Hebei	2000–2001	ELISA^g^	IgG or CAg positive	No	75	43 (57.33)	9
Yu et al. [53]	2006	Beijing		ELISA^c^	IgG positive	Gender, age	128	18 (14.06)	9
Dubey et al. [35]	2007	Guangzhou, Guangdong	2006	MAT	≥ 1:40	No	34	27 (79.41)	6
Yu et al. [51]	2008	Beijing	1999–2005	ELISA^c^	IgG positive	Gender, age	335	50 (14.93)	9
Huang et al. [38]	2008	Haikou, Hainan	2007–2008	ELISA^d^	IgG positive	No	251	14 (5.58)	8
Zhang et al. [56]	2009	Guangzhou, Guangdong		ELISA^d^	IgG positive	Stray or pet, gender, age	206	52 (25.24)	9
Sun et al. [46]	2009	Beijing and neighbor	2008	ELISA^c^	IgG positive	Gender	172	32 (18.60)	8
Zhang et al. [64]	2009	Beijiang, Xinjiang		IHA^a^	≥ 1:64	No	42	3 (7.14)	8
Lu et al. [40]	2010	Huhehaote, Inner Monglia	2009–2010	ELISA^e^	IgG positive	Gender	87	9 (10.34)	9
Lu et al. [65]	2010	Lanzhou, Gansu	2008–2009	Test Paper^k^	antigen positive	Age	159	14 (8.81)	8
Xie et al. [50]	2010	Shenzhen, Guangdong	2009–2010	ELISA^d^	IgG positive	No	278	13 (4.68)	9
Zhang et al. [55]	2010	Zhengzhou, Henan	2009	IHA^a^	≥ 1:64	Gender, age	58	9 (15.52)	10
Chen et al. [62]	2010	Shanghai	2009–2009	IHA^a^	≥ 1:64	Stray or pet	270	65 (24.07)	9
Qian et al. [45]	2010	Beijing		ELISA^h^	IgG positive	Stray or pet	323	58 (17.96)	8
Huang et al. [37]	2011	Zhejiang		Test Paper^k^	antigen positive	Stray or pet	341	91 (26.69)	10
Wu et al. [49]	2011	Lanzhou, Gansu	2010–2011	MAT	≥ 1:25	Stray or pet, Gender, age	221	47 (21.27)	10
Wang et al. [48]	2012	Shanghai	2010–2011	ELISA^d^	IgG positive	Gender, age	145	25 (17.24)	10
Qian et al. [44]	2012	Beijing	2009–2011	MAT	≥ 1:20	No	64	37 (57.81)	8
Qi et al. [43]	2012	Beijing	2011	IHA^a^	≥ 1:64	No	176	7 (3.98)	9
Wang et al. [47]	2012	Zhengzhou, Henan	2010–2011	IHA^a^	≥ 1:64	Age	195	102 (52.31)	10
Cui et al. [63]	2012	Beijing	2010–2011	ELISA^c^	IgG positive	Age, gender, season	561	119 (21.21)	8
Yu et al. [52]	2013	Pudong, Shanghai		ELISA^c^	IgG positive	Stray or pet	27	5 (18.52)	8
Zhuo et al. [60]	2013	Taizhou, Jiangsu	2012	IHA^a^	≥ 1:64	No	215	43 (20.00)	9
Wang et al. [69]	2013	Fujian	2012	ELISA^d^	IgG positive	No	530	238 (45.00)	7
Liu et al. [39]	2014	Zhenjiang, Jiangsu	2013	ELISA^d^	IgG positive	Stray or pet	116	24 (20.69)	10
Deng et al. [34]	2014	Changsha, Hunan	2011–2012	ELISA^c^	IgG positive	Gender, age	75	21 (28.00)	10
Fu et al. [67]	2014	Xuzhou, Jiangsu	2010–2012	ELISA^c^	IgG positive	No	41	17 (41.46)	8
Zhao et al. [57]	2015	Beijing	2012–2014	ELISA^c^	IgG positive	Season	286	60 (20.98)	9
Deng et al. [33]	2015	Shanghai	2014	ELISA^i^	IgG positive	No	91	5 (5.49)	8
Lai et al. [68]	2015	Beijing	2013	ELISA^i^	IgG positive	No	48	2 (4.17)	8
Mayilai et al. [42]	2015	Kuche, Xinjiang	2014	ELISA^j^	IgG positive	Gender, age	87	34 (39.08)	8
Zheng et al. [59]	2015	Shandong	2012–2013	ELISA^e^	IgG positive	Gender, age	589	23 (3.90)	10
Cong et al. [61]	2016	Lanzhou, Gansu	2014–2015	MAT	≥ 1:25	Stray or pet, Gender, age	362	70 (19.34)	10

*Abbreviations*: *ELISA* Enzyme Linked Immunosorbent Assay, *IHA* Indirect Haemagglutination test, *MAT* Modified Agglutination Test, Test Paper, test paper for TOXO-Ag

^a^The test kits were produced by Lanzhou Veterinary Research Institute, Chinese Academy of Agricultural Science (Cut-off titer 1:64)

^b^By Shanghai No. 2 Medical School, Parasite Research Section (Cut-off titer 1:80)

^c^By Zhuhai S.E.Z Haitai Biological Pharmaceuticals Co., Ltd. (IgG positive)

^d^By Shenzhen Combined Biotech Co., Ltd. (IgG positive)

^e^By Shanghai Touching Technology Co., Ltd. (IgG positive)

^f^By Hubei Academy of Medical Sciences (IgG or CAg positive)

^g^By Zhejiang Institute of Parasitic Disease (IgG or CAg positive)

^h^By Animal Medicine College, China Agricultural University (IgG positive)

^i^By French ID-VET company (IgG positive)

^j^By Parasite Laboratory of Xinjiang Agricultural University (IgG positive)

^k^By Quicking Biotech Co., Ltd. (antigen positive)

### Pooling and heterogeneity analyses

**Fig. 2:**
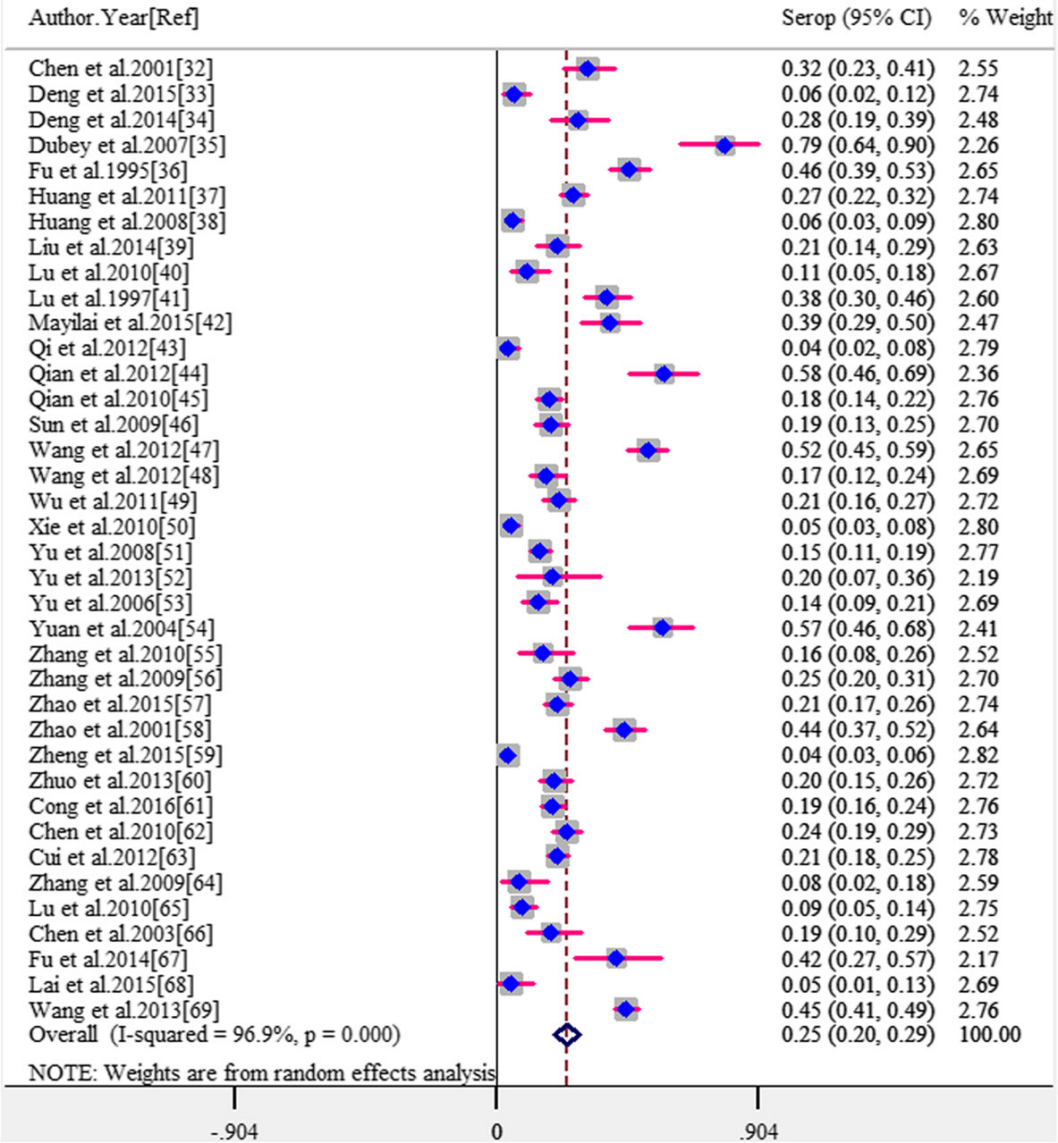
Forest plot of the seroprevalence estimates of *T. gondii* in cats with random-effects analyses

The seroprevalence estimates of *T. gondii* in cats are shown in a forest plot (Fig. 2). *Toxoplasma gondii* seroprevalence of each study varied from 3.9 to 79.4% (median 20.3%) with substantial heterogeneity among studies (*χ* ^2^ = 1,192.78, *P* < 0.001; I^2^ = 96.9%, 95% CI: 96.1–97.7). The pooled overall seroprevalence was 24.5% (95% CI: 20.1–29.0) when calculated using the random-effects model.

**Fig. 3:**
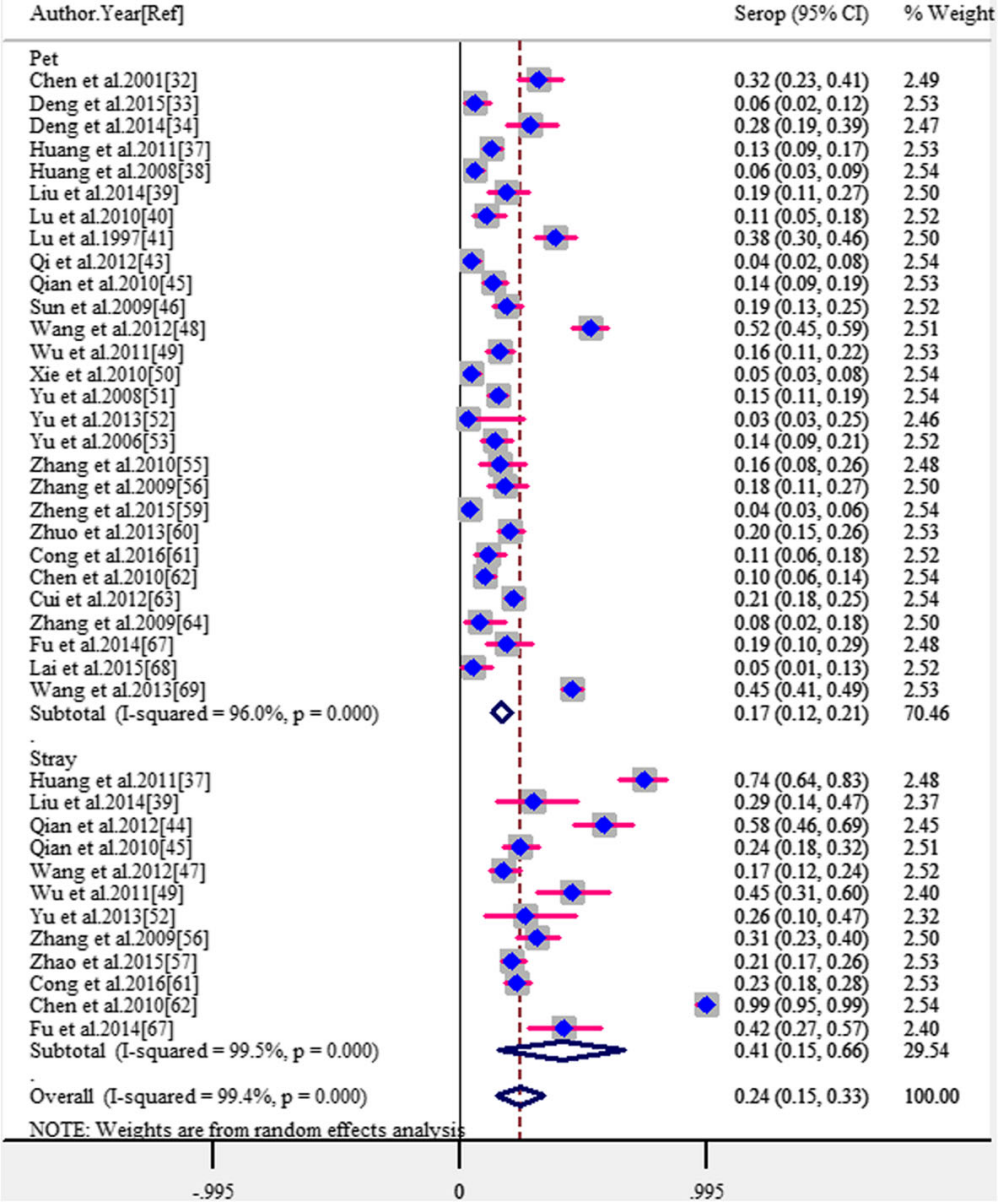
Forest plot of the seroprevalence estimates of *T. gondii* in cats by stray or pet cats

**Fig. 4:**
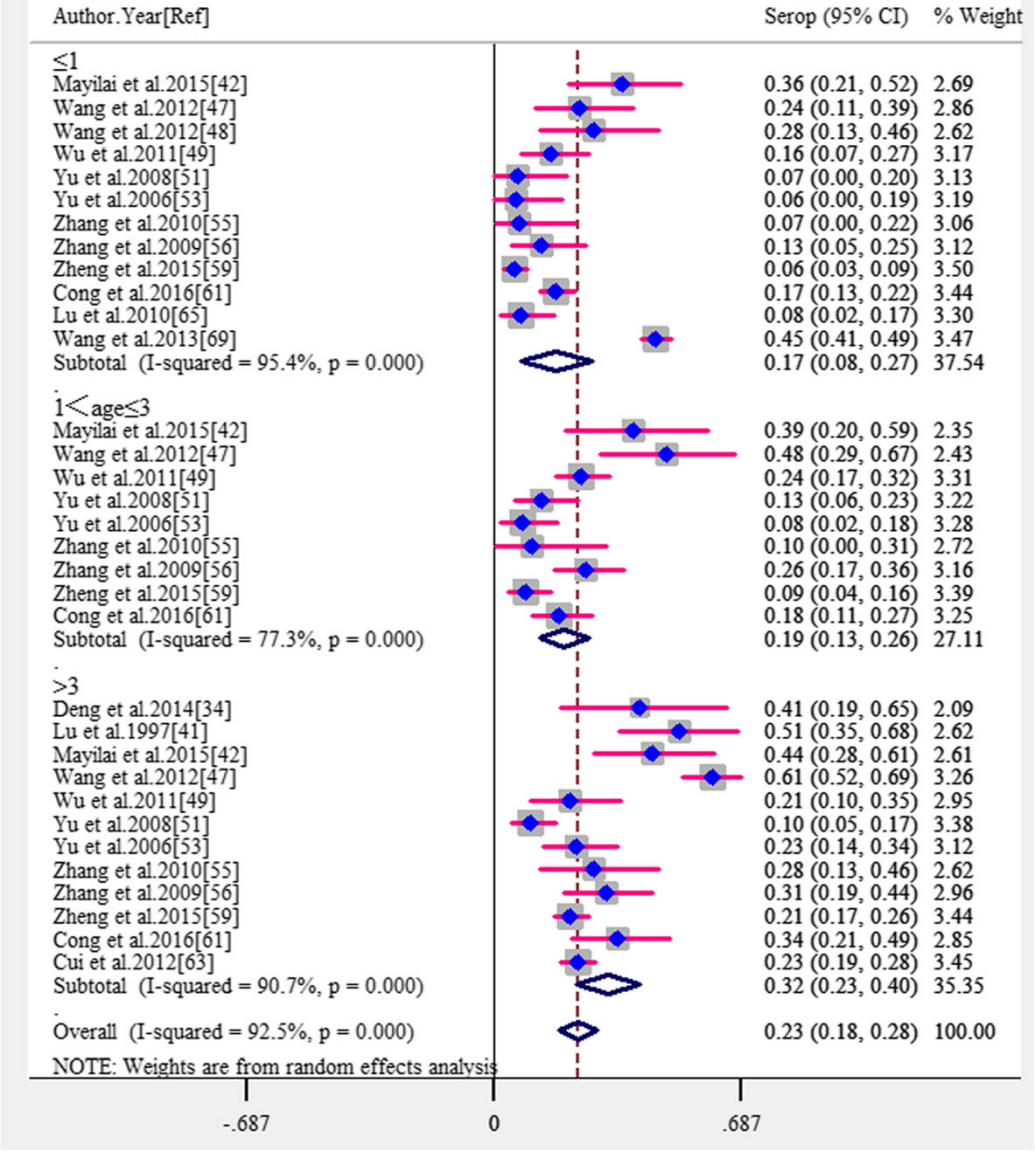
Forest plot of the seroprevalence estimates of *T. gondii* in cats by age groups (≤ 1 year-old, ≤ 3 year-old or > 3 year-old) with random-effects analyses

The pooled estimates by potential risk factors are presented in Table 2. In subgroup analyses, because there was a significantly high level of heterogeneity among studies within most subgroups, all estimates of the pooled seroprevalence for each subgroup were calculated using the random-effects model. Of the 38 studies, 33 provided information on the groups of cats investigated (12 about stray cats and 28 about pet cats), and the pooled seroprevalence was significantly higher in stray cats (40.9%) than in pet cats (16.7%) (*P* < 0.001; OR = 3.00; 95% CI: 1.60–5.64) (Fig. 3). In the eight studies which presented data from both stray and pet cats, the seroprevelance was also significantly higher in stray cats (35.9%) than in pet cats (13.0%) (*P* < 0.001; OR = 4.87; 95% CI: 2.10–11.30). The seroprevalence varied from 17.4 to 31.6% among three age groups (Fig. 4) and the difference was also significant (*P* < 0.05), as seen in Table 2. A total of 13 studies provided estimates about gender, but no significant difference was observed between male and female cats (*P* = 0.743; OR = 1.07; 95% CI: 0.67–1.65). Similarly, no significant difference was observed among survey seasons (*P* = 0.911) as detailed in Table 2. On the basis of geographical regions, the lowest seroprevalence (17.4%) was in Western China and the highest (32.3%) was in Central China but with no significant difference among regions (*P* = 0.469). When stratified according to the main serological test used, no significant difference was found among the three main methods (*P* = 0.109), as see in Table 2. Two studies applied a different method (i.e. Test paper) to screen *T. gondii* antigens in cats’ serum. After excluding these two studies, the pooled seroprevalence and its 95% CI were 24.9% (95% CI: 20.3–29.0), which was closely aligned with the previous estimates.

**Table 2:** Pooled estimates of *T. gondii* in cats by potential risk factors with meta-analysis

Factors related to *T. gondii* seroprevalence in cats		No. of studies included	No. of positive cats	Total no. of cats	Pooled seroprevalence (95% CI)	Heterogeneity			Meta-regression	
						Q (*χ* ^2^)	Q-P	I^2^(%)	*P*-value	OR (95% CI)
Overall		38	1,650	7,285	0.245 (0.201–0.290)	1,192.78	< 0.001	96.90		
Group	Stray	12	400	1,261	0.409 (0.154–0.664)	2,020.66	< 0.001	99.50	**0.001**	3.00 (1.60–5.64)
	Pet	28	958	5,284	0.167 (0.124– 0.209)	676.81	< 0.001	96.00		Reference
Gender	Male	14	261	1,333	0.212 (0.170–0.255)	42.46	< 0.001	71.70	0.743	1.07 (0.67–1.65)
	Female	14	225	1,196	0.200 (0.156–0.244)	45.8	< 0.001	73.80		Reference
Age	Y > 3	12	318	1,115	0.316 (0.229–0.403)	118	< 0.001	90.70	**0.018**	2.77 (1.39–5.53)
	1 < Y ≤ 3	9	102	523	0.195 (0.127–0.263)	35.23	< 0.001	77.30		1. 54 (0.72–3.30)
	Y ≤ 1	12	335	1,249	0.174 (0.076–0.272)	238.78	< 0.001	95.40		Reference
Survey season	Spring	3	87	335	0.282 (0.181–0.384)	8.47	0.014	76.40	0.911	1.15 (0.46–2.92)
	Summer	3	58	259	0.226 (0.175–0.276)	0.93	0.628	0		0.90 (0.34–2.36)
	Autumn	3	45	219	0.249 (0.099–0.398)	10.37	0.006	80.70		0.91 (0.34–2.43)
	Winter	3	43	176	0.247 (0.206–0.289)	1.06	0.589	0		Reference
Region	Eastern	28	1,308	5,894	0.249 (0.197–0.302)	1,010.27	< 0.001	97.30	0.469	1.43 (0.54–3.79)
	Central	4	165	433	0.323 (0.161–0.484)	41.86	< 0.001	92.80		2.35 (0.58–9.45)
	Western	6	177	958	0.174 (0.105–0.243)	42.41	< 0.001	88.20		Reference
Serological test	ELISA	22	895	4,556	0.207 (0.155–0.259)	619.24	< 0.001	96.60	0.109	0.29 (0.10–1.08)
	IHA	10	469	1,548	0.272 (0.157–0.388)	323.63	< 0.001	97.20		0.44 (0.13–1.50)
	MAT	4	181	681	0.432 (0.229,0.635)	99.79	< 0.001	97.00		Reference

*Abbreviations: 95% CI* 95% confidence interval, *ELISA* Enzyme Linked Immunosorbent Assay, I^2^, the inconsistency index describing the percentage of variability due to heterogeneity rather than sampling error; *IHA* Indirect Haemagglutination test, *MAT* Modified Agglutination Test, *Q* Cochran’s Q-tests for heterogeneity, *Q-P* p-value of Q-tests

The figures in bold are for a significant difference between/among subgroups with Meta-regression at the level of 0.05

### Bias

**Fig. 5:**
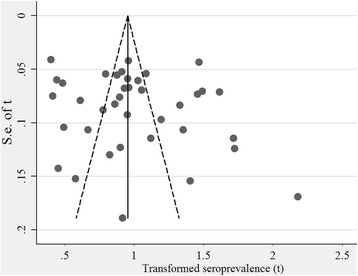
Funnel plots of the arcsine transformed seroprevalence estimates (t) of *T. gondii* in cats *Abbreviation*: se, standard error

The funnel plots showed no publication bias (see Fig. 5), which was also confirmed from Egger’s test (the bias coefficients b = 2.49; 95% CI: -7.25–9.23; *t* = 1.06, *P* = 0.294). No theoretical missing study was filled by the Duval and Tweedie non-parametric method (see Additional file 2).

### Sensitivity tests

The sensitivity tests indicated that all single-study-omitted estimates lay within the 95% CI of the respective overall seroprevalence (see Additional file 2). This suggested that the pooled seroprevalence was not substantially influenced by any single study. The stability of such results validated the rationality and reliability of our analyses.

## Discussion

In this study, we searched five databases and identified a total of 38 relevant articles which contained eligible data on the seroprevalence of *T. gondii* in 7,285 cats across mainland China. To our knowledge, this is the first study to assess the national level of *T. gondii* seroprevalence in cats, which given the intimate relationship between cats and humans and the consequences of *T. gondii* infections in pregnant women and immunocompromised people, could be of great importance to public health and associated control measures. The overall seroprevalence of *T. gondii* in cats in mainland China from 1991 to 2015 was 24.5% (95% CI: 20.1–29.0). Although comparable with the prevalence recorded in Spain (25.5% in pet cats and 36.9% in stray cats) [70] and much lower than in Ethiopia (87.72%) [71] and Estonia (60.8%) [72], it was much higher than in the neighbouring country Japan (5.4% in pet cats) [73, 74]. Our study shows a moderate seroprevalence of *T. gondii* in cats in mainland China when compared to the average seroprevalence of 30–40% worldwide [1]. In our research two factors (stray or domestic, and cat age) were significantly associated with *T. gondii* seroprevalence.

There was high heterogeneity in seroprevalence levels in cats across mainland China among the eligible studies, but no significant publication bias was found at our cut-off level of 0.05 with either Egger’s test, or Duval-Tweedie’s method. This high heterogeneity index is suggestive of potential variations, which could be due to real characteristics of cats surveyed, geographical regions, surveyed seasons or due to study effects such as diagnostic methods. To trace the source of heterogeneity, cats were first divided into two subgroups, stray cats or pet cats. In stray cats the pooled seroprevalence of *T. gondii* infection was significantly higher than in pet cats. This is consistent with studies reported in Spain [70], Tehran [75] and Brazil [76]. This higher seroprevalence in stray cats may be associated with their hunting and diet habits, as a stray cat lives outdoors, hunts and potentially feeds on oocyst contaminated scraps and garbage and/or *Toxoplasma*-infected wild birds and rodents, with more risk of ingestion of the parasite. Although the seroprevalence in pet cats is lower than in strays, nearly 1 in 5 pet cats has been exposed to *T. gondii* and the number of pet cats is rapidly increasing in China, strongly associated with the rapid social change of the country [21]. Some practices such as feeding pet cats raw meat may increase the chance of exposure to *T. gondii* and transmission from them [20].

The seroprevalence in cats increased with cat age, ranging from 17.4% in cats ≤ 1 years of age in comparison with 31.6% in cats > 3 years of age. This agrees with a study in which a significantly higher seroprevalence of *T. gondii* is observed in an adult cat group compared with the juveniles [77, 78]. This is likely to be explained by the positive association between an increase in age, with an increased risk of exposure to *T. gondii* oocysts over time, and a long lived immune response to this. No significant difference was observed between sexes, again supporting a study which showed that sex was not considered a determining factor for infection with *T. gondii* in cats [49]. This indicates that there is little or no difference between the cat sexes in both infection risk behaviour and/or immunological susceptibility.

*Toxoplasma gondii* is widely distributed, especially in warm, moist and low altitude regions [79], and at temperate to tropical temperatures oocysts remain infectious for up to 1.5 years [80]. Thus, it would be predicted that infections in cats may differ among regions or seasons in relation to climate [81]. Indeed, after the data were stratified based on geographical regions, cats in Central China including Hunan, Hubei and Henan provinces, characterized by a subtropical monsoon climate and suitable for the survival and sporulation of oocysts in the wild, had a higher seroprevalence than in other regions although this was not significant in the overall analyses. This is partly due to a low number of studies from the central region. In terms of seasons in which surveys were conducted, the highest pooled seroprevalence in cats was in Spring and the lowest in Summer, but this was also not significant, again likely due to small sample sizes within the studies (i.e. 176 to 335 cats per subgroup).

Although the serological methods to identify *T. gondii* infection differed among studies, ELISA, IHA and MAT were the most common and there were no significant differences among these methods in the reported seroprevalences. In testing seroprevalence of *T. gondii* in cats with ELISA, IHA and LAT (Latex Agglutination Test), the results from these three kits were similar [74]. By using MAT and ELISA in detecting *T. gondii* in cats, no significant difference was seen between the two methods [82]. All three diagnostic methods were also compared for the routine screening of *T. gondii* infections and were shown to have good compliance with each other [83]. All of these findings, including our meta-regression analysis here and meta-analyses on the adjusted seroprevalence with both sensitivity and specificity of each test (see Additional file 3; and original data, see Additional file 4), suggest that testing method was unlikely to be a significant source of heterogeneity in this analysis.

There are two main limitations in our meta-analysis. First, as the numbers of eligible studies in subgroups are small, the estimates and the predictive values of the risk factors should be assessed accordingly. Secondly, no information about cats’ environment, such as rural or urban areas, has been described, thus making it impossible to assess the effect of this potentially important factor with regard to implementing control. However, this is the first study, to our knowledge, to estimate the overall seroprevalence of *T. gondii* in cats in mainland China, leading the way for future research in areas and cat groups which might be informative for future control interventions if required. In addition, there was no information on potentially important issues such as: (i) are pet cats allowed to go outside? and (ii) the effect of rural *versus* urban areas on *T. gondii* seroprevalence levels. Future studies incorporating the potential differences between urban and rural areas are required if we are to reduce overall infection levels in China.

## Conclusions

The seroprevalence of *T. gondii* in cats in mainland China was moderate (up to 24%) and associated with cats’ activities (i.e. stray or pet cats) and cat age. However, due to the increasing ownership of pet cats in China and the intimate association between cats and humans, particularly with China’s large susceptible population, and nearly 1 on 5 pet cats being *T. gondii* seropositive this might present a significant exposure risk to cat owners. Therefore, in order to reduce the infections of *T. gondii* in humans *via* cat contacts (or/and eating raw meat) and the environmental contamination with *T. gondii* oocysts by stray or pet cats, approaches such as educational programs on the potential risk of *T. gondii* when raising cats, improvement in personal hygiene, and good pet-keeping management should be recommended.

## Additional files

Additional file 1: Checklist of items to include when reporting a meta-analysis. (DOC 73 kb)
Additional file 2: Filled funnel plot by the Duval & Tweedie method and sensitivity analyses. (DOC 73 kb)
Additional file 3: Meta-analyses on the seroprevalence of *T. gondii* in cats adjusted with sensitivity and specificity of each test. (DOC 188 kb)
Additional file 4: The data generated from each included article during this study. (DOC 242 kb)

## Abbreviations

CI: Confidence intervals
CBM: Chinese biomedical literature database
CNKI: China national knowledge infrastructure
ELISA: Enzyme linked immunosorbent assay
IHA/IHAT: Indirect haemagglutination test
MAT: Modified agglutination test
OR: Odds ratio
PRISMA: Preferred reporting items for systematic reviews and meta-analyses
