# Supplementary material for: A systematic review and meta-analysis of the seroprevalence of Toxoplasma gondii in cats in mainland China
Source: Parasit Vectors. 2017 Jan 13;10:27. doi: 10.1186/s13071-017-1970-6 (PMC5237326; doi:10.1186/s13071-017-1970-6)
Supplement: Additional file 2: — Filled funnel plot by the Duval & Tweedie method and sensitivity analyses. (DOC 73 kb) [file 13071_2017_1970_MOESM2_ESM.doc]

**Additional file 2. Filled funnel plot by the Duval and Tweedie method and sensitivity analyses**


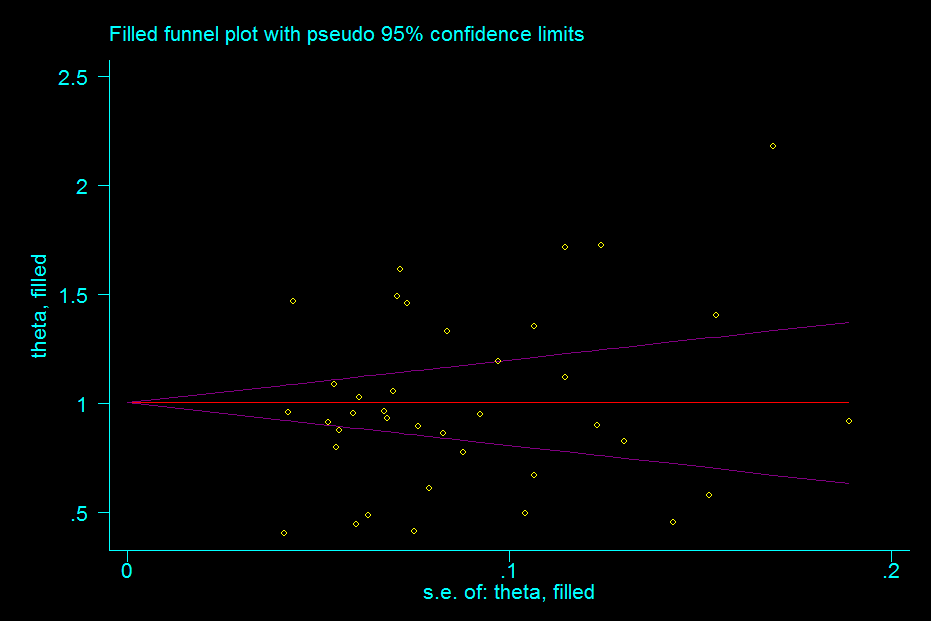


**Figure S2.1** Filled funnel plot by the Duval and Tweedie non-parametric method


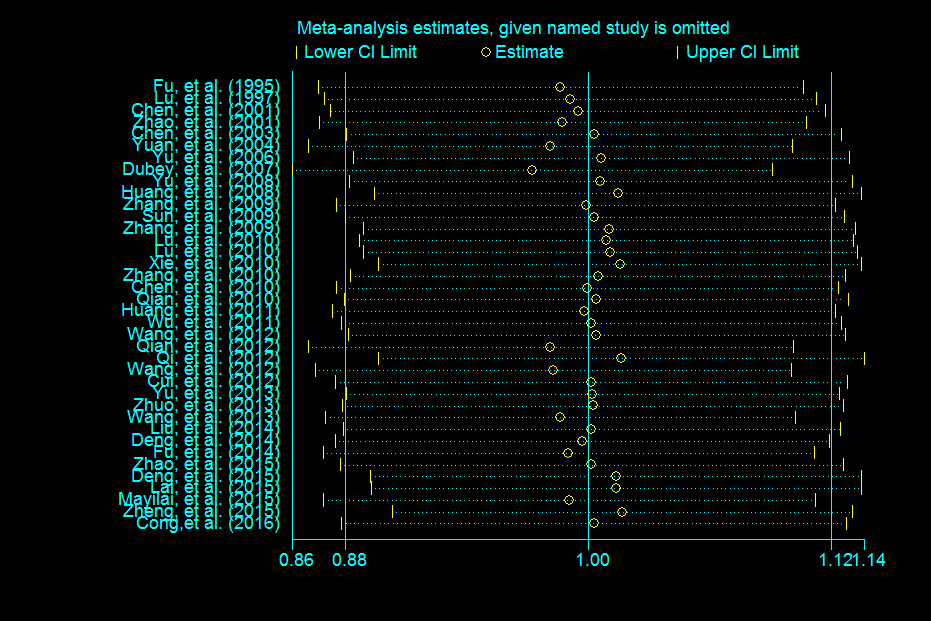


**Figure S2.2** Sensitivity analyses with Meta-analysis random-effect estimates
