# Supplementary material for: A systematic review and meta-analysis of the seroprevalence of Toxoplasma gondii in cats in mainland China
Source: Parasit Vectors. 2017 Jan 13;10:27. doi: 10.1186/s13071-017-1970-6 (PMC5237326; doi:10.1186/s13071-017-1970-6)
Supplement: Additional file 3: — Meta-analyses on the seroprevalence of T. gondii in cats adjusted with sensitivity and specificity of each test. (DOC 188 kb) [file 13071_2017_1970_MOESM3_ESM.doc]

**Additional file 3. Meta-analyses on the seroprevalence of *T. gondii* in cats adjusted with sensitivity and specificity of each test**

As the specificity and the sensitivity for *T. gondii* may differ among different serological methods, we employed the following formula [1] to calculate the adjusted seroprevalence (t) :

p_Adj_ = [original seroprevalence – (1 – Sp)] / [Se – (1 – Sp)]

The specificity and sensitivity for ELISA was 92.6% and 96.5% respectively [2], for IHA 89.8% and 96.6% respectively [3], for MAT 96.2% and 98% respectively [4], and for Test Paper both 100% [5].

For every study and all studies combined Fig. S3.1 present the non-adjusted seroprevalence and Fig. S3.2 the adjusted seroprevalence (see below Fig. S3.1 and Fig. S3.2). The results showed no obvious difference between both. We then performed subgroup meta-analyses based on the potential risk factors and results are shown below in Table S3.1 and Table S3.2. Again, no overt difference was seen between the adjusted and the non-adjusted.


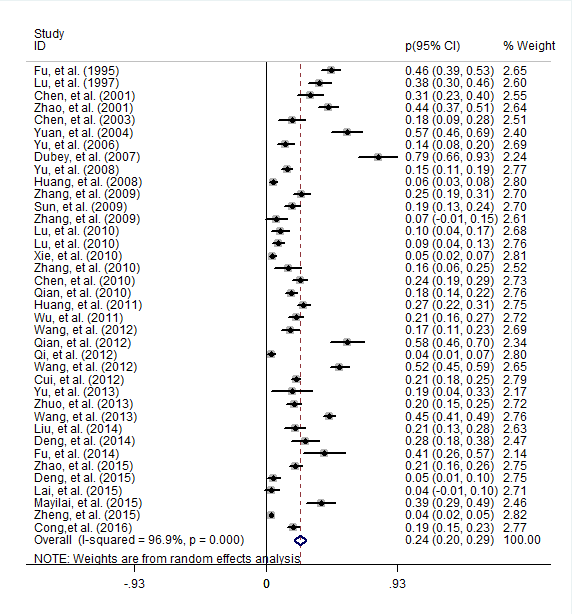


**Figure S3.1** Forest plot of the seroprevalence of *T. gondii* in cats with random-effects analyses


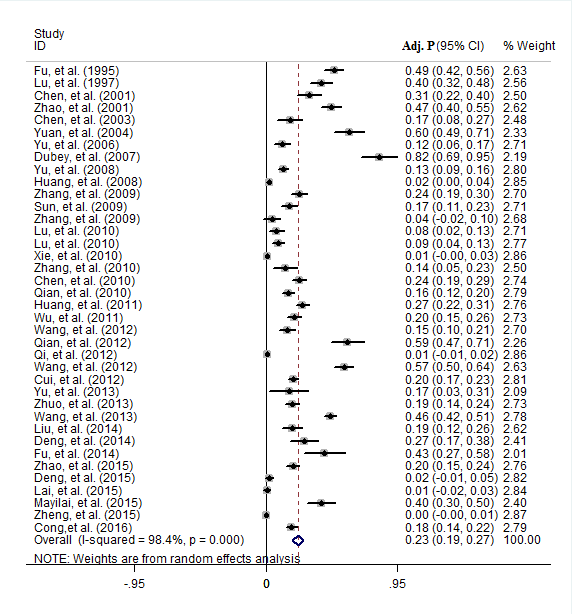


**Figure S3.2** Forest plot of the adjusted seroprevalence of *T. gondii* in cats with random-effects analyses

**Table S3.1 Pooled seroprevalence by potential risk factors with random-effects model**

| Risk factors |  | No. of studies | No. positive cats | No. total cats | Seroprevalence (95%CI) | Heterogeneity, I2 |
| --- | --- | --- | --- | --- | --- | --- |
|
| Overall | | 38 | 1650 | 7285 | 0.243(0.198,0.288) | 96.90% |
| Group | Stray | 12 | 400 | 1261 | 0.350(0.252,0.447) | 93.10% |
|  | Pet | 28 | 958 | 5284 | 0.168(0.125, 0.211) | 96.20% |
| Gender | Male | 14 | 261 | 1333 | 0.208(0.166,0.250) | 70.90% |
|  | Female | 14 | 225 | 1196 | 0.195(0.151,0.239) | 73.40% |
| Age | Y>3 | 12 | 318 | 1115 | 0.316(0.229,0.403) | 90.70% |
|  | 1<Y≤3 | 9 | 102 | 523 | 0.186(0.117,0.255) | 77.80% |
|  | Y≤1 | 12 | 335 | 1249 | 0.164(0.066,0.263) | 95.60% |
| Survey season | Spring | 3 | 87 | 335 | 0.280(0.179,0.381) | 75.70% |
|  | Summer | 3 | 58 | 259 | 0.222(0.172,0.273) | 0 |
|  | Autumn | 3 | 45 | 219 | 0.243(0.095,0.392) | 79.70% |
|  | Winter | 3 | 43 | 176 | 0.242(0.179,0.305) | 0 |
| Region | Eastern | 28 | 1308 | 5894 | 0.247(0.195,0.299 ) | 97.30% |
|  | Central | 4 | 165 | 433 | 0.320(0.155,0.485) | 93.00% |
|  | Western | 6 | 177 | 958 | 0.171(0.100,0.241) | 88.60% |
| Serological test | ELISA | 22 | 895 | 4556 | 0.205(0.153,0.257) | 96.60% |
|  | IHA | 10 | 469 | 1548 | 0.270(0.153,0.386) | 97.30% |
|  | MAT | 4 | 181 | 681 | 0.434(0.230,0.637) | 97.00% |

**Table S3.2 Pooled adjusted seroprevalence by potential risk factors with random-effects model**

| Risk factors |  | No. of studies | No. positive cats | No. total cats | True Seroprevalence (95%CI) | Heterogeneity, I2 |
| --- | --- | --- | --- | --- | --- | --- |
|
| Overall | | 38 | 1650 | 7285 | 0.233(0.194,0.272) | 98.40% |
| Group | Stray | 12 | 400 | 1261 | 0.347(0.246,0.447) | 93.80% |
|  | Pet | 28 | 958 | 5284 | 0.150(0.115, 0.185) | 97.80% |
| Gender | Male | 14 | 261 | 1333 | 0.198(0.151,0.246) | 79.40% |
|  | Female | 14 | 225 | 1196 | 0.183(0.134,0.231) | 80.90% |
| Age | Y>3 | 12 | 318 | 1115 | 0.318(0.215,0.421) | 93.80% |
|  | 1<Y≤3 | 9 | 102 | 523 | 0.173(0.097,0.248) | 85.40% |
|  | Y≤1 | 12 | 335 | 1249 | 0.148(0.053,0.243) | 97.20% |
| Survey season | Spring | 3 | 87 | 335 | 0.283(0.164,0.401) | 82.80% |
|  | Summer | 3 | 58 | 259 | 0.211(0.161,0.260) | 0 |
|  | Autumn | 3 | 45 | 219 | 0.241(0.070,0.421) | 85.40% |
|  | Winter | 3 | 43 | 176 | 0.233(0.171,0.295) | 0 |
| Region | Eastern | 28 | 1308 | 5894 | 0.235(0.192,0.278 ) | 98.50% |
|  | Central | 4 | 165 | 433 | 0.325(0.132,0.519) | 95.10% |
|  | Western | 6 | 177 | 958 | 0.159(0.085,0.232) | 90.80% |
| Serological test | ELISA | 22 | 895 | 4556 | 0.187(0.142,0.231) | 98.10% |
|  | IHA | 10 | 469 | 1548 | 0.272(0.130,0.414) | 98.70% |
|  | MAT | 4 | 181 | 681 | 0.442(0.222,0.661) | 97.50% |

***Reference***

1. Tu XM, Litvak E, Pagano M. Issues in human immunodeficiency virus (HIV) screening programs. American Journal of Epidemiology. 1992; 136:244-55.

2. Dabritz HA, Gardner IA, Miller MA, Lappin MR, Atwill ER, Packham AE, Melli AC, Conrad PA. Evaluation of two *Toxoplasma gondii* serologic tests used in a serosurvey of domestic cats in California. Journal of Parasitology. 2007; 93:806-16.

3. Yang YX, Chen YK, Wei SJ, Song RH. Efficiency of three methods for detecting *Toxoplasma* IgG antibody. Chinese Journal of Schistosomiasis Control. 2014; 26:109-10.

4. Mainar-Jaime RC, Barberán M. Evaluation of the diagnostic accuracy of the modified agglutination test (MAT) and an indirect ELISA for the detection of serum antibodies against *Toxoplasma gondii* in sheep through Bayesian approaches**.** Inter Nationes; 1983.

5. Wang YH, Ke sheng LI, Cai XP, Hui fang DU, Xue rui LI, Zhang DL. Development of colloidal gold test strip for rapid detection of *Toxoplasma* antibody. Veterinary Science in China. 2007.
