# Supplementary material for: A systematic review and meta-analysis of the seroprevalence of Toxoplasma gondii in cats in mainland China
Source: Parasit Vectors. 2017 Jan 13;10:27. doi: 10.1186/s13071-017-1970-6 (PMC5237326; doi:10.1186/s13071-017-1970-6)
Supplement: Additional file 4: — The data generated from each included article during this study. (DOC 242 kb) [file 13071_2017_1970_MOESM4_ESM.doc]

**Additional file 4. The data generated from each included article during this study**

| **Article included** | **No. cats examined** | **No. cats positive (%)** | **Region** | **Group** | | **Cat’s age group** | | | **Season** | | | | **Gender** | | **Test methods** |
| --- | --- | --- | --- | --- | --- | --- | --- | --- | --- | --- | --- | --- | --- | --- | --- |
|  |  |  |  | Stray cats | Pet cats | ≤1 year-old | 1year-old<age≤3 year-old | age>3 year-old | Spring | Summer | Autumn | Winter | Male | Female |  |
|  | 200 | 92(46.00) | Eastern |  |  |  |  |  |  |  |  |  |  |  | IHA |
|  | 142 | 54(38.01) | Eastern |  | 54/142 | 37/109 | | 17/33 | 22/52 | 9/31 | 16/38 | 7/21 | 31/78 | 23/64 | IHA |
|  | 105 | 33(31.43) | Central |  | 33/105 |  |  |  |  |  |  |  |  |  | ELISA |
|  | 185 | 82(44.32) | Eastern |  |  |  |  |  |  |  |  |  |  |  | IHA |
|  | 65 | 12(18.46) | Eastern |  | 12/65 |  |  |  |  |  |  |  |  |  | IHA |
|  | 75 | 43(57.33) | Eastern |  |  |  |  |  |  |  |  |  |  |  | ELISA |
|  | 128 | 18(14.06) | Eastern |  | 18/128 | 1/23 | 3/43 | 14/62 |  |  |  |  | 8/54 | 10/74 | ELISA |
|  | 34 | 27(79.41) | Eastern |  |  |  |  |  |  |  |  |  |  |  | MAT |
|  | 335 | 50(14.93) | Eastern |  | 50/335 | 1/21 | 7/55 | 9/92 |  |  |  |  | 16/106 | 10/81 | ELISA |
|  | 251 | 14(5.58) | Eastern |  | 14/251 |  |  |  |  |  |  |  |  |  | ELISA |
|  | 206 | 52(25.24) | Eastern | 36/117 | 16/89 | 5/40 | 19/75 | 16/52 |  |  |  |  | 18/82 | 22/85 | ELISA |
|  | 172 | 32(18.60) | Eastern |  | 32/172 |  |  |  |  |  |  |  |  |  | ELISA |
|  | 42 | 3(7.14) | Western |  | 3/42 |  |  |  |  |  |  |  |  |  | IHA |
|  | 87 | 9(10.34) | Western |  | 9/87 |  |  |  |  |  |  |  | 5/26 | 3/30 | ELISA |
|  | 159 | 14(8.81) | Western |  |  | 3/45 | 11/114 | |  |  |  |  |  |  | Test Paper |
|  | 278 | 13(4.68) | Eastern |  | 13/278 |  |  |  |  |  |  |  |  |  | ELISA |
|  | 58 | 9(15.52) | Central |  | 9/58 | 1/19 | 1/13 | 7/26 |  |  |  |  | 5/31 | 4/27 | IHA |
|  | 270 | 65(24.07) | Eastern | 43/43 | 22/227 |  |  |  |  |  |  |  |  |  | IHA |
|  | 323 | 58(17.96) | Eastern | 33/137 | 25/186 |  |  |  |  |  |  |  |  |  | ELISA |
|  | 341 | 91(26.69) | Eastern | 58/78 | 33/263 |  |  |  |  |  |  |  |  |  | Test Paper |
|  | 221 | 47(21.27) | Western | 19/42 | 28/179 | 8/53 | 31/129 | 8/39 |  |  |  |  | 26/104 | 21/117 | MAT |
|  | 145 | 25(17.24) | Eastern | 25/145 |  | 7/26 | 18/119 | |  |  |  |  | 14/78 | 11/67 | ELISA |
|  | 64 | 37(57.81) | Eastern | 37/64 |  |  |  |  |  |  |  |  |  |  | MAT |
|  | 176 | 7(3.98) | Eastern |  | 7/176 |  |  |  |  |  |  |  |  |  | IHA |
|  | 195 | 102(52.31) | Central |  | 102/195 | 8/35 | 12/25 | 82/135 |  |  |  |  |  |  | IHA |
|  | 561 | 119(21.21) | Eastern |  | 119/561 | 14/101 | | 71/307 | 33/128 | 36/169 | 23/150 | 27/114 | 44/188 | 23/124 | ELISA |
|  | 27 | 5(18.52) | Eastern | 5/20 | 0/7 |  |  |  |  |  |  |  |  |  | ELISA |
|  | 215 | 43(20.00) | Eastern |  | 43/215 |  |  |  |  |  |  |  |  |  | IHA |
|  | 530 | 238(45.00) | Eastern |  | 238/530 | 238/530 |  |  |  |  |  |  |  |  | ELISA |
|  | 116 | 24(20.69) | Eastern | 8/28 | 16/88 |  |  |  |  |  |  |  |  |  | ELISA |
|  | 75 | 21(28.00) | Central |  | 21/75 | 15/60 | | 6/15 |  |  |  |  | 9/33 | 12/42 | ELISA |
|  | 41 | 17(41.46) | Eastern | 17/41 |  |  | |  |  |  |  |  |  |  | ELISA |
|  | 91 | 5(5.49) | Eastern |  | 5/91 |  |  |  |  |  |  |  |  |  | ELISA |
|  | 286 | 60(20.98) | Eastern | 60/286 |  |  |  |  | 32/155 | 13/59 | 6/31 | 9/41 |  |  | ELISA |
|  | 2 | 48(4.17) | Eastern |  | 2/48 |  |  |  |  |  |  |  |  |  | ELISA |
|  | 87 | 34(39.08) | Western |  |  | 12/34 | 8/21 | 14/32 |  |  |  |  | 11/31 | 23/56 | ELISA |
|  | 589 | 23(3.90) | Eastern |  | 23/589 | 10/184 | 7/83 | 59/287 |  |  |  |  | 36/311 | 31/278 | ELISA |
|  | 362 | 70(19.34) | Western | 59/260 | 11/102 | 41/239 | 14/79 | 15/44 |  |  |  |  | 38/211 | 32/151 | MAT |

**Reference**

1. Fu B, Zhao YQ, Yang BJ, Han GD, Shao QF, Li JM, et al. Epidemiological investigations on toxoplasmosis in ShanDong province. Chin J Parasit Dis Cont. 1995; 08:205-7.

2. Lu Q, Huang AM. Seroprevalence of *Toxoplasma gondii* infection in outpatient cats in Shanghai. Chin J Zoonoses. 1997; 13:69-70..

3. Chen CY. Study on *Toxoplasma gondii* infection of pets and population. Chin J Zoonoses. 2001; 17:76-7.

4. Zhao YQ, Zhen TM, Wang JX, Fu B, Han GD. A survey of *Toxoplasma gondii* infection in animals in Shandong province. J Prev Med Inf. 2001; 17:185-.

5. Chen HR, Huang BJ. The report of toxoplasma serological survey. In: Small animal medicine. Haebing, Heilongjiang; 2003, p.181-182.

6. Yuan WY, Ma K, Yang HL. A survey of *Toxoplasma gondii* infection in animals in Hebei province. Chin J Schist Cont. 2004; 16:72,6.

7. Yu YL, Fu LJ, Wang M. Seroprevalence of *Toxoplasma gondii* infection in dogs and cats in Beijing areas. Chin J Vet Med. 2006; 42:7-9.8. Dubey JP, Zhu XQ, Sundar N, Zhang H, Kwok OC, Su C. Genetic and biologic characterization of *Toxoplasma gondii* isolates of cats from China. Vet Parasitol. 2007; 145:352-6.

9. Yu JH, Ding J, Xia ZF, Lin DG, Li YL, Jia JY, Liu Q. Seroepidemiology of *Toxoplasma gondii* infection in pet dogs and cats in Beijing, China. Acta Parasitol. 2008; 53:317-9. 10. Huang SM, Zhou QP, Cui K, Huang ZS, Li QX. Analysis of *Toxoplasma gondii* infection in pet dogs and cats in HaiKou City. Chin Trop Med. 2008; 8:1462,392.

11. Zhang H, Zhou DH, Zhou P, Lun ZR, Chen XG, Lin RQ, et al. Seroprevalence of *Toxoplasma gondii* infection in stray and household cats in Guangzhou, China. Zoonoses Public Health. 2009; 56:502–5.

12. Sun X, Li RH, Sun HY, Zhao DM. Epidemiology survey of *Toxoplasma gondii* infection in pet dogs and pet cats in Beijing and surrounding regions. Heilongjiang Anim Sci Vet Med. 2009:110.

13. Zhang Y, Liu F, Xu X, He L, Li A, Zhang X. Serological investigation of toxoplasmosis in Beijiang district, Xinjiang province. Herb Magazine. 2009:22-4.

14. Lu AT, Gao Y, Du S. Survey on cats and dogs infected with *Toxoplasma gondii* at part area of Inner Mongolia. Anim Husb Feed Sci. 2010; 31:155-6.

15. Lu WY, Ha L, Cao LP, Yang LM, Xue YP. The current prevalence of toxoplasmosis in dogs and cats in Lanzhou and other five cities (states). Anim Husb Vet Med. 2010; 42:109.

16. Xie GP, Geng YJ, Zhang RL, Huang DN, Gao ST, Zhang Q, et al. Epidemiology survey of *Toxoplasma gondii* infection and related factors in pet dogs and domestic cats in Shenzhen. Chin Trop Med. 2010; 10:1075-7.

17. Zhang H, Li P, Chai J. Epidemiology survey of toxoplasmosis in dogs and cats in Zhengzhou urban district. Heilongjiang Anim Sci Vet Med. 2010:74-5.

18. Chen J. Epidemiology survey of *Toxoplasma gondii* in pet cats and dogs in Shanghai. Shanghai: Shanghai Jiaotong University; 2010.

19. Qian WF, Yu SS, Wang H, Shan D, Liu Q. Epidemiology survey of toxoplasmosis and isolation and characterization of *Toxoplasma gondii* strains from dogs and cats. In: Proceedings of the 2011 Annual Conference of Chinese Institute of Animal Husbandry and Veterinary. Chengdu. 2011: 718.

20. Huang LQ, Li H, He SG, Mao HR. Epidemiology survey of *Toxoplasma gondii* infection in dogs, cats and pigs in ZheJiang areas. Chin J Vet Med. 2011; 47:39-41.

21. Wu SM, Zhu XQ, Zhou DH, Fu BQ, Chen J, Yang JF, et al. Seroprevalence of *Toxoplasma gondii* infection in household and stray cats in Lanzhou, northwest China. Parasit Vectors. 2011; 4:214-7.

22. Wang Q, Jiang W, Chen YJ, Liu CY, Shi JL, Li XT. Prevalence of *Toxoplasma gondii* antibodies, circulating antigens and DNA in stray cats in Shanghai, China. Parasit Vectors. 2012; 5:190.

23. Qian W, Hui W, Su C, Dan S, Xia C, Na Y, et al. Isolation and characterization of *Toxoplasma gondii* strains from stray cats revealed a single genotype in Beijing, China. Vet Parasitol. 2012; 187:408-13.

24. Qi HX, Zhang HY, Zhang WJ, Zhang XM, Cao YZ. Epidemiology survey of toxoplasmosis in pets (dogs, cats ). Beijing Agricult J. 2012:70-1.

25. Wang HY, Pei SL, Hao ZF, Zhou M. Epidemiology survey of toxoplasmosis in pets in Zhengzhou. Henan J Agricult Sci. 2012; 41:153-4.

26. Cui LL, Yu YL, Liu S, Wang BB, Zhang ZX, Wang M. The epidemiology survey of *toxoplasmosa gondii* in cats and dogs in Beijing. Chin J Vet. 2012; 48:7-10.

27. Yu XQ, Wen DL, Gong DD, Zhang ZH, Gong JW, Sun FH, et al. Seroprevalence survey and analysis of toxoplasmosis in dogs and cats in Pudong New Area. Shanghai J Anim Husb Vet Med. 2013:36-7.

28. Zhuo GR, Di HS, Lu W, Liu JD, Zhang H, Wang CF. Analysis of serum antibody of *Toxoplasma gondii* in cats in Taizhou area with different detection methods. Jiangsu Agricult Sci. 2013; 41:190-1.

29. Wang K. The survey of infection of *Toxoplasma gondii* in some animals and genetying of swine in Fujian. Fujian: Fujian Agriculture and Forestry University; 2013.

30. Liu QX, Wang S, Wang LQ, Xing J, Gao WJ, Liu GF, et al. Seroprevalence of *Toxoplasma gondii* infection in dogs and cats in Zhenjiang City, Eastern China. Asian Pac J Trop Biomed. 2014; 4:725-8.

31. Deng GQ, Ou LY, Tang XM, Wang WG. Seroprevalence of *Toxoplasma gondii* infection in cats and dogs in urban area of Changsha. Chin J Vet. 2014; 50:74-5.

32. Fu LL, Yan C, Liu ZZ, Kong DL, Lv L, Shi N. Isolation and identification of *Toxoplasma gondii* strains from cats in Xuzhou region. Chin J Schist Cont. 2014; 26:656-7.

33. Deng B, Ge J, Yang XC, Li KH, Zhou JP. Seroprevalence of *toxoplasma gondii* infection in domestic dogs and cats in urban area of Shanghai in 2014. Shanghai J Anim Husb and Vet Med. 2015:44-5.

34. Zhao J, Cang YJ, Wang X. Investigation of *Toxoplasma gondii* infection in stray cats and wild animals in Beijing zoo. J Wildlife. 2015; 36:270-4.

35. Lai PG, Li MW, Zhai XJ, Tian HY. The epidemiological survey of some zoonosis in pet cats and dogs in Beijing city. Chin J Vet Med. 2015; 51:69-70.

36. Mayilai, Chen QL, Liu ML, Tuersun, Bayinzhahan. Investigation of *Toxoplasma gondii* infection in dogs, cats in KuChe areas. Xinjiang J Anim Husb. 2015:25-7.

37. Zheng SY, Zheng F, Li SP, Duan XM, Geng JF, Jia HH. Epidemiology survey of toxoplasmosis in dogs and cats in parts of Shandong province. Chin J Vet Med. 2015; 51:74-5.

38. Cong W, Meng QF, Blaga R, Villena I, Zhu XQ, Qian AD. *Toxoplasma gondii*, *Dirofilaria immitis*, *feline immunodeficiency virus* (FIV), and *feline leukemia virus* (FeLV) infections in stray and pet cats (Felis catus) in northwest China: co-infections and risk factors. Parasitol Res. 2016; 115:217-23.
